# Dynamic Regulation of Peroxisomes and Mitochondria during Fungal Development

**DOI:** 10.3390/jof6040302

**Published:** 2020-11-20

**Authors:** Raful Navarro-Espíndola, Fernando Suaste-Olmos, Leonardo Peraza-Reyes

**Affiliations:** Departamento de Bioquímica y Biología Estructural, Instituto de Fisiología Celular, Universidad Nacional Autónoma de México, Mexico City 04510, Mexico; rasane18@gmail.com (R.N.-E.); fsuaste@ifc.unam.mx (F.S.-O.)

**Keywords:** mitochondria, peroxisome, fungi, cell differentiation, sexual development, organelle dynamics, peroxisome fission, mitochondrial fission, mitophagy, pexophagy

## Abstract

Peroxisomes and mitochondria are organelles that perform major functions in the cell and whose activity is very closely associated. In fungi, the function of these organelles is critical for many developmental processes. Recent studies have disclosed that, additionally, fungal development comprises a dynamic regulation of the activity of these organelles, which involves a developmental regulation of organelle assembly, as well as a dynamic modulation of the abundance, distribution, and morphology of these organelles. Furthermore, for many of these processes, the dynamics of peroxisomes and mitochondria are governed by common factors. Notably, intense research has revealed that the process that drives the division of mitochondria and peroxisomes contributes to several developmental processes—including the formation of asexual spores, the differentiation of infective structures by pathogenic fungi, and sexual development—and that these processes rely on selective removal of these organelles via autophagy. Furthermore, evidence has been obtained suggesting a coordinated regulation of organelle assembly and dynamics during development and supporting the existence of regulatory systems controlling fungal development in response to mitochondrial activity. Gathered information underscores an important role for mitochondrial and peroxisome dynamics in fungal development and suggests that this process involves the concerted activity of these organelles.

## 1. Introduction

Eukaryotic cells are composed of distinct membrane-bound organelles, which provide compartmentalization for the different tasks of a cell. The composition and activity of these subcellular structures are regulated during cell development to cope with the specific developmental cell demands. Research in recent years has demonstrated that the developmental regulation of organelle activity includes a precise modulation of their intracellular dynamics, which involves changes in their abundance, morphology, and arrangement. Furthermore, cell development also relies on mechanisms that control the distribution of organelles within cells, establishing their appropriate location at their sites of action, and regulating their distribution and partitioning during cell division and differentiation. Moreover, organelles establish dynamic crosstalk and interactions, which allow them to coordinate their activity and to collectively drive diverse complex cellular processes. Peroxisomes and mitochondria provide a fascinating example of the cooperative interplay that organelles maintain to perform, regulate, and coordinate their functions. These organelles have long been known to be required for important developmental processes of fungi, and mounting evidence has recently disclosed that the developmental modulation of their dynamics can also be determinant for fungal development. Moreover, emerging evidence has also revealed that fungal developmental processes might involve a concerted regulation of peroxisome and mitochondrial functioning and dynamics. Here we review recent findings on the regulation of peroxisome and mitochondrial activity and dynamics during fungal development, with emphasis on the developmental processes of mycelial fungi.

### 1.1. Peroxisomes and Mitochondria Are Closely Interrelated Organelles

Peroxisomes and mitochondria are organelles that perform fundamental functions in the cell and that maintain a very close association [1]. Early since their initial characterization, peroxisomes were observed to be frequently located near mitochondria [2,3], hinting to a close functional association between them. Recent evidence has shown that peroxisomes physically interact with mitochondria at defined strategic sites via specific tether proteins and that these interactions are important for the regulation of their activity, crosstalk, and dynamics [4,5,6,7,8,9]. In correlation with their spatial association, peroxisomes and mitochondria maintain an intimate metabolic interplay. These organelles share several metabolic enzymes and cooperatively drive important metabolic pathways, notably the fatty acid β-oxidation pathway and the glyoxylate shunt [10,11,12]. Peroxisomes and mitochondria have a prominent role in the formation and disposal of reactive oxygen species (ROS) [13,14]. These organelles participate in redox metabolism and homeostasis, and they can collectively contribute to cellular redox signaling. Peroxisomes and mitochondria maintain redox communication, which can modulate their activity and define redox-driven cell developmental outcomes [13,15,16]. In addition, peroxisomes and mitochondria share signaling proteins (e.g., [17,18]), and they can serve as signaling platforms that collectively integrate complex signaling pathways, such as the innate immune signaling that drives the immune response during infection by viruses and intracellular bacteria [17,19,20,21].

The processes that govern the biogenesis and dynamics of peroxisomes and mitochondria are also closely interrelated. The transcriptional regulation of peroxisome and mitochondrial biogenesis is controlled by common factors in mammals [1,22], and the sorting of some of their proteins involves the simultaneous action of the protein import machinery of both organelles [23]. Moreover, peroxisome biogenesis relies on mitochondrial-derived vesicles, which fuse with vesicles originated on the endoplasmic reticulum (ER) to produce peroxisome precursors, competent to develop into mature peroxisomes [24]. In addition, shared fission machinery drives the division of both peroxisomes and mitochondria throughout eukaryotes [25], and the processes conducting their selective elimination via autophagy—referred to as pexophagy and mitophagy, respectively—are also interrelated [26].

### 1.2. Common Mechanisms Regulate Peroxisome and Mitochondrial Dynamics: An Overview on the Division and Autophagic Removal of Peroxisomes and Mitochondria

**Figure 1:**
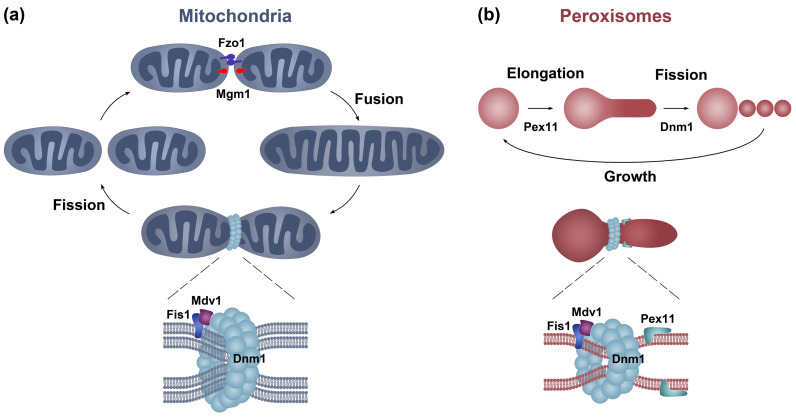
Mitochondrial and peroxisome division in fungi. (**a**) The mitochondrial arrangement is determined by fusion and fission events that are mediated by dynamin proteins. Fusion is driven by the dynamins Fzo1 (mitofusin) and Mgm1 (Opa1) at the mitochondrial outer and inner membranes, respectively, while Dnm1 mediates fission. (**b**) Peroxisomes multiply by growth and division from preexisting organelles, in a process that also involves Dnm1. Dnm1 is recruited to mitochondria and peroxisomes by the membrane receptor Fis1 through interactions with the Mdv1 adapter (lower panels). Dnm1 then assembles into spirals around the organelles, which constrict and sever their membranes upon GTP hydrolysis. Peroxisome fission is preceded by the organelle elongation, which is promoted by the membrane elongation factor Pex11. This protein also serves as a Dnm1 activator.

Peroxisomes and mitochondria are highly dynamic organelles whose dynamics are regulated in response to distinct environmental, metabolic, and developmental cues. Mitochondria compose dynamic networks, which undergo constant remodeling involving fusion and fission events. The balance between these processes defines mitochondria arrangement and connectivity and is driven by conserved dynamin GTPases (Figure 1a) [27]. Mitochondrial fusion consists of the sequential fusion of the outer and inner mitochondrial membranes, which are mediated by mitofusins (Mfn1/2/Fzo1) and Optic Atrophy 1 (OPA1/Mgm1), respectively. Mitochondrial fission is conducted by dynamin-related-protein 1 (Drp1/Dnm1), which is recruited to mitochondria by membrane receptor proteins, like Fis1 in fungi (along with the adapter Mdv1), or Mff, MiD49, and MiD51 in mammals. Under specific physiologic conditions, Fis1 also contributes to mitochondrial fission in mammals (reviewed in [27,28]). The sites of mitochondrial fission are defined by physical contacts between mitochondria and the ER, which mediate an initial actin dynamics-dependent constriction and facilitates the assembly of the fission machinery [29,30,31]. Interestingly, mitochondria-ER contact sites also define the site of assembly of the mitochondria fusion machinery and serve to modulate mitochondrial morphology by spatially and bidirectionally coordinating the fission and fusion events [32].

Although no fusion is known to occur between mature peroxisomes, these organelles share with mitochondria the proteins that govern their fission (Figure 1) [25]. Throughout eukaryotes, peroxisome fission is mediated by Dnm1/Drp1, which is recruited to this organelle also by the membrane receptors Fis1 (along with the Mdv1 adapters) (Figure 1b) and Mff in fungi and mammals, respectively. In addition, a second dynamin-like protein—Vps1p—can mediate peroxisome fission in the yeast *Saccharomyces cerevisiae*. Peroxisome fission is preceded by the elongation of the peroxisome membrane via Pex11 family proteins, which also participate in the recruitment of the fission machinery and the activation of Dnm1 GTPase [9,33].

The autophagic elimination of peroxisomes and mitochondria are also interrelated processes. Autophagy is a conserved process that directs cytoplasmic components to the vacuole (lysosome in animals) for degradation [34]. The most prominent autophagic degradation system—macroautophagy (hereafter referred to as autophagy)—consists of the assembly of a double membrane-bound organelle, the autophagosome, which results from the formation and elongation of an isolation membrane (or phagophore) that encloses portions of cytoplasm destined to degradation. Following their closure, autophagosomes fuse with vacuoles to deliver their contents for digestion [34,35]. Bulk autophagy non-selectively removes portions of the cytoplasm. In contrast, numerous selective autophagic systems specifically target and remove defined cargoes, like portions of the ER (ER-phagy), peroxisomes (pexophagy), and mitochondria (mitophagy) [36]. Selective autophagy relies on autophagic receptors, which recognize specific cargoes and connect them to the protein complexes that promote phagophore formation and expansion, facilitating autophagosome assembly around a given cargo [37]. Different autophagic receptors target peroxisomes and mitochondria. In yeasts, the autophagy-related protein (Atg) Atg32 constitutes the mitophagy receptor (along with Atg33 in *S. cerevisiae*) [38,39], whereas Atg36 (or Atg30 in the yeast *Pichia pastoris*) provide the pexophagy receptors [40,41]. These receptors converge on the selective autophagy adapter Atg11, a scaffold protein that recruits early-acting autophagic proteins to promote autophagosome formation around specific cargoes [37,42]. In addition, the receptors also bind to Atg8, which is a core autophagic machinery component that promotes phagophore elongation [37,43]. Atg11 also forms a complex with the sorting nexins Atg20 and Atg24 (also known as Snx42 and Snx4, respectively), which are also required for selective autophagy, notably of peroxisomes and mitochondria, and likely participate in phagophore membrane remodeling [42]. Interestingly, consistent with coordinated regulation of mitophagy and pexophagy, common signaling factors regulate these processes both in mammals [44,45,46,47] and in yeasts [48,49].

## 2. Peroxisomes and Mitochondria Are Required for Fungal Development

Mitochondria constitute the main source for energy production in aerobic eukaryotes, perform a central role in intermediary metabolism, and provide a metabolic hub for the synthesis of precursors of major cell biosynthetic pathways. Additionally, mitochondria participate in distinct cell signaling processes and are involved in the regulation of cell differentiation [50,51,52]. In keeping with the essential role of mitochondria in the cell, defects in the biogenesis of this organelle, which do not result in lethality, compromise the developmental processes conducting both asexual and sexual reproduction in fungi [53,54,55]. Likewise, as illustrated in the model ascomycetes *Neurospora crassa* and *Podospora anserina*, the reproductive developmental processes of these fungi are compromised when there are deficiencies in the mitochondrial respiratory chain. Both asexual sporulation (conidiation) and sexual development of *N. crassa*, as well as sexual development of *P. anserina*—a fungus that reproduces exclusively sexually—are impaired by defects in the activity of the respiratory complex I, which is not essential for vegetative growth due to presence of alternative NADH dehydrogenases [56,57,58,59,60]. Similarly, these developmental processes are impaired by the defective activity of the respiratory complexes III or IV, where respiration is sustained by an alternative oxidase [61,62,63,64]. Moreover, some of these respiratory deficiencies disturbed fungal development without drastically affecting vegetative growth, disclosing specific bioenergetic requirements for fungal developmental processes, and suggesting a developmental regulation of energy metabolism. In addition, mitochondria function is determinant for fungal aging [65,66] and pathogenesis, including the virulence of important human pathogenic fungi [67,68,69,70].

Peroxisomes also play important roles during fungal development. The analyses of different peroxisome biogenesis factors (peroxins, denoted by the Pex acronym) have shown that defects in the formation of these organelles have important repercussions in asexual developmental processes, like conidiation and the yeast-hyphal morphogenic transition of some dimorphic fungi. Similarly, distinct peroxisome biogenesis defects have important detrimental effects in the development of the infective structures of pathogenic fungi, like appressoria [71,72]. Additionally, along with mitochondria, peroxisomes are required for the synthesis of toxins associated with pathogenic development [73,74,75,76]. Moreover, peroxisomes perform multiple roles in the developmental processes conducting sexual reproduction in fungi, including the formation of sexual fructifications, the induction, and progression of meiotic development, and the maturation and germination of sexual spores [77]. The central peroxisome metabolic pathways—like the fatty acid β-oxidation pathway and the glyoxylate cycle—have been associated with some of these developmental processes. Nonetheless, many functions performed by peroxisomes during fungal development remain undisclosed. Emerging evidence from diverse fungi has recently shown that, in addition to the specific functions performed by peroxisomes and mitochondria per se, the regulation of their activity and intracellular dynamics are also determinant for many developmental processes in fungi.

## 3. Peroxisome and Mitochondrial Dynamics Are Involved in Asexual Development in Fungi

**Figure 2:**
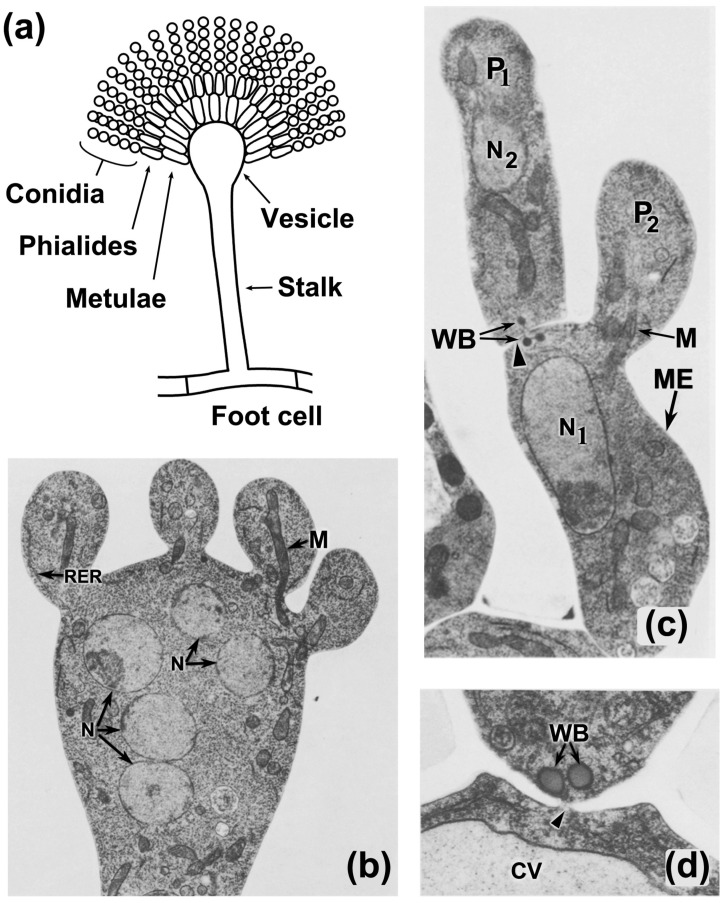
Subcellular organization during *Aspergillus nidulans* conidiation. (**a**) Schematics of an *A. nidulans* conidiophore. Asexual spore (conidium) formation in *A. nidulans* takes place on specialized aerial conidiophores, which consist of a large specialized hypha—the stalk—that grows away from the substrate and swells at the tip to produce a conidiophore vesicle. This vesicle produces numerous primary sterigmata (metulae) by budding, which, in turn, differentiate secondary sporogenous sterigmata (phialides) at their apices. Conidia emerge by budding from the tip of these specialized cells. The stalk extends from a specialized foot cell, which anchors the conidiophore and connects it to the substratum mycelium. (**b**) Ultrastructural analyses of a conidiophore vesicle showing four young metulae. At this stage, mitochondria (M) and rough ER (RER) strands extend into metulae, while nuclei (N) remain positioned below metulae. (**c**) Metula (ME) has differentiated a phialide (P1) delimited by a septum and that is developing a second phialide (P2). Visible is a mitochondrion (M) extending into the developing phialide, and Woronin bodies (WB) bordering the septum (arrowhead) at the base of the first phialide. (**d**) Mature septum at the base of a metula. Note the Woronin bodies bordering the pore (arrowhead) of the septum; CV, conidiophore vesicle. (**b**–**d**) Adapted from [79], with permission.

Hyphae are the fundamental cell structure of filamentous fungi, able to differentiate into very specialized cells and to develop complex tissues during fungal development [78]. During asexual development, vegetative hyphae differentiate specialized sporogenous cells that produce spores in several different ways. In most Dikarya fungi, specialized hyphal structures (conidiophores) produce conidia, which are the main form of asexual dispersal in most of these fungi (Figure 2a illustrates the structure of a representative conidiophore). Moreover, hyphal cells are also able to differentiate highly specialized structures that allow fungi to successfully develop diverse lifestyles. These structures include specialized infection structures that allow predatory and pathogenic fungi to trap and invade their hosts.

### 3.1. Mitochondrial and Peroxisome Dynamics Are Regulated during Asexual Development

As illustrated in *A. nidulans*, early ultrastructural cytological analyses of conidiation showed that the differentiation of the cell types of this developmental process is accompanied by the orderly segregation of organelles (Figure 2), including mitochondria [79], supporting the existence of a regulatory system controlling the accuracy of this process. Mechanisms controlling the accurate segregation of organelles have been uncovered for the budding process of *S. cerevisiae*. In this yeast, the cell division cycle involves a coordinated and regulated partitioning of peroxisomes and mitochondria between mother and daughter cells [80]. The formation of protein tethers at the mother cell cortex facilitates organelle retention, whereas the association with the actin-dependent myosin V motor Myo2 promotes traffic to the daughter cell of both peroxisomes and mitochondria [80]. Peroxisomes anchor on the mother cell cortex by forming a tether between Inp1 and peroxisomal Pex3 [81,82,83,84]. Organelle fission facilitates traffic by the subsequent attachment to Myo2, mediated by Inp2 [85]. Comparable interactions take place with mitochondria. The mitochondria-ER-cortex anchor (MECA) is a complex comprising the proteins Num1 and Mdm36 [86,87,88], which alongside the protein Mfb1 [89], form two separate tethers that retain mitochondria in the mother cell. The traffic of mitochondria into the bud needs Myo2, Mmr1, and Ypt11 [90,91,92,93]. When peroxisomes or mitochondria arrive at the bud tip, Inp1-Pex3 [85], and Mmr1 [94,95] tethers, respectively, anchor organelles at the bud. These mechanisms assure an even partitioning of peroxisomes and mitochondria in both cells after budding in *S. cerevisiae.*

Early evidence for developmental regulation of peroxisome dynamics associated with a specific cell differentiation process was disclosed in the nematophagous fungus *Arthrobotrys oligospora*. This fungus develops complex tridimensional adhesive hyphal networks to trap nematodes during its predacious development. The differentiation of these trap cells is accompanied by a proliferation of electron-dense microbodies, which are different from peroxisomes present in vegetative hyphae and likely represent specialized peroxisomes, as revealed by the cytochemical detection of D-amino-acid oxidase and catalase in their matrix. These unusual peroxisomes originated at a very early stage of trap development, and they mainly emerged from specialized regions of the ER [96,97]. Interestingly, while the number of these microbodies increased during trap cell maturation, the amount of ER and mitochondria in these cells decreased. Furthermore, while mature trap cells are crowded with electron-dense microbodies, the number of these organelles decreased in nematode-penetrating hyphae upon infection, in a process that involved their vacuolar sequestration and degradation, which likely constitutes their selective elimination by pexophagy [96,97,98]. The proliferation of these specialized peroxisomes presumably plays a role in trap cell differentiation or the initial stages of nematode infection. It has recently been shown that the elimination of the protein StuA in *A. oligospora*—a transcription factor of the APSES protein family that is indispensable for trap formation—decreases the transcription levels of genes involved in peroxisome biogenesis and proliferation, and results in loss of peroxisomes [99], showing that peroxisome proliferation is part of the developmental program that controls trap cell formation in *A. oligospora.*

### 3.2. Peroxisome and Mitochondria Dynamics Are Required for Asexual Sporulation

Mutants of the peroxisome-mitochondrial fission machinery in fungi generally have alterations in mycelial growth; although, the extent of these alterations can vary among species, and depending on the available nutrients, denoting different contributions of the fission machinery in hyphal growth [100,101,102,103,104,105,106,107,108,109]. Moreover, the morphology of mitochondria and peroxisomes in mutants defective for the mitochondrial-peroxisome fission proteins can also vary. Consistent with participation in mitochondrial fission, the elimination of these proteins among different fungi results in undivided, often elongated mitochondria. However, large spherical mitochondria, which are interconnected by thin tubules, can also be produced upon loss of the fission proteins, as revealed in *Aspergillus fumigatus* [104]. Moreover, in *P. anserina* distinct mitochondrial arrangements exhibiting differential distribution along hyphae are produced when the fission machinery is absent. In this case, mainly elongated mitochondria in the apical region are followed by large spherical mitochondria in subapical segments, which are embedded in a mitochondrial network that exhibits a highly dense packaging towards the distal regions [102]. These observations imply different hyphal regional constraints implicated in the regulation of mitochondrial dynamics. Of note, in addition to the expected peroxisome elongation produced by deletion of *FIS1* or *DNM1* in *P. anserina*, the segregation of peroxisomes during the ramification of hyphae is also disturbed when these genes are missing, indicating that the loss of the fission machinery has repercussions in the dynamics of peroxisomes beyond their division process [102].

Asexual sporulation in most studied fungi is disturbed by defects in the peroxisome-mitochondrial fission machinery. The elimination of proteins of the fission machinery results in decreased conidia formation in diverse ascomycete fungi, differing in their lifestyle and evolutionary lineage, including the blast rice fungus *Magnaporthe oryzae* [105,108,110], the human pathogen *A. fumigatus* [104], the saprophytic *A. nidulans* [100,101] and the entomopathogenic *Metarhizium robertsii* [107]. Likewise, the formation of the different asexual spores—i.e., conidia and blastospores—produced by entomopathogen *Beauveria bassiana* is also affected by the elimination of Dnm1, Fis1, or Mdv1 [106].

In some of these fungi, like *M. oryzae* [110] and *A. nidulans* [100], these conidiation deficiencies are associated with a reduced number of conidiophores and a decrease in the number of spores produced per conidiophore. In contrast, in *B. bassiana* conidiation appears to be only delayed [106], indicating different contributions for the peroxisome-mitochondria fission machinery to fungal conidiation. Of interest, despite these conidiation deficiencies, mitochondria can be segregated into conidia in *Aspergilli* even when the fission proteins are absent [100,104]. Likewise, albeit microconidia in *P. anserina* rather function as male gametes during the sexual cycle (also known as spermatia), the segregation of mitochondria into these specialized conidia is also not affected upon loss of Fis1 or Dnm1 [102]. These observations indicate that a Dnm1-independent division mechanism operates during conidiogenesis.

There remains ambiguity about the precise contribution of the fission machinery to conidiation. Evidence from *Aspergilli* support a decreased mitochondrial respiratory activity when the peroxisome-mitochondrial fission machinery is absent [100,104], and a moderate reduction of mycelial ATP generation occurs after *Fis1, Mdv1*, or *Dnm1* deletion in *B. bassiana* [106], suggesting that a reduced generation of energy in mitochondria contribute to the conidiation defects of the fission mutants. Mitochondria and peroxisomes constitute an important source for ROS in the cell. ROS play important roles in the regulation of cell differentiation in fungi [111] and compromised fission of these organelles could lead to deregulation in ROS formation and redox homeostasis. Dysfunction of the fission machinery led to increased sensitivity to H_2_O_2_ in *M. oryzae* [108], as well as to increased mitochondrial ROS production and reduced tolerance to menadione-produced oxidative stress in *A. nidulans*, suggesting an increased sensitivity to mitochondrial-derived ROS in this fungus [100]. These observations suggest that increased ROS formation is associated with the developmental defects of the peroxisome-mitochondrial fission mutants.

Mitochondrial fission and fusion antagonistically regulate the organization of the mitochondrial network [112]. Consistent with a prominent role for mitochondria fission in conidiation, the conidiation defects of *A. fumigatus* fission mutants can be partially alleviated upon the elimination of the mitochondrial fusion protein Mgm1 in these mutants [104]. Of note, fusion machinery components in *A. fumigatus* are essential, suggesting that mitochondrial fusion is more important than fission for the overall physiology of this fungus. On the other hand, the fission process is required for the selective degradation of peroxisomes and mitochondria [113,114]; therefore, some developmental deficiencies of the fission mutants could be related to a poor degradation of both organelles by selective autophagy (Section 7). Altogether, the above observations indicate that the peroxisome and mitochondrial division process is required for multiple processes involved in fungal conidiation.

## 4. Peroxisome and Mitochondrial Dynamics Are Required for Pathogenic Development in Fungi

Appressoria have been recently described as a widespread structure in Dikarya saprotrophic filamentous fungi, enabling them to penetrate solid surfaces [115]. Nevertheless, it is in pathogenic fungi where appressoria are the main structure developed to achieve infection of plants or insects by the penetration of the host cuticle [116,117]. In these fungi, conidia or blastospores adhere to the leaf or insect cuticle upon recognition of physical cues, like hydrophobicity and surface hardness. A germ tube then emerges from these spores and flattens at the tip, where the appressorium differentiates. This process requires precise cell cycle control and involves the autophagic degradation of the spore intracellular contents, which are then channeled into the developing appressorium. Fungi may develop appressoria as simple germ-tube terminal swellings or as complex dome-shaped differentiated structures. Alternatively, some fungi develop multicellular penetration structures called infection cushions. Upon infection, appressoria undergo a polarization rearrangement and develop a penetration peg, where mechanical pressure is responsible for cuticle rupture and posterior hyphal invasion. Some fungi need melanin for appressoria functionality, whereas others use it only as a structural component [117]. On the other side, cell morphogenic transitions are essential for virulence in human pathogenic fungi. Species of this latter kind tend to be dimorphic or even polymorphic, transiting between multiple morphologies to better adapt to their environment and to effectively invade and disperse in their hosts. In many cases, the invasiveness of this fungi depends on the differentiation of infective hyphae [118]. The fission and fusion machinery have been related to the differentiation and development of infection structures, and are required to achieve full virulence in several fungi.

Entomopathogenic fungi lose virulence capacity in absence of the core fission gene *Dnm1* [107] or of the fission adapter-encoding genes *Fis1* and *Mdv1* [106]. Nevertheless, *M. robertsii* can develop appressoria in absence of these genes; still, whether their functionality is affected remains to be resolved.

In *M. oryzae*, the fission machinery is needed for this phytopathogenic fungus to achieve full virulence, as mutants for any of its three components show decreased virulence [105,108,110]. Appressoria develop in these strains but their functionality is compromised. The competency of these structures to penetrate host plants is highly reduced, and the growth of the invasive hyphae inside the plant is limited [105,108]. Consequently, the lesions produced by these strains are less severe than those produced by wild-type strains. Moreover, the fission machinery is involved in turgor generation, cell wall integrity, and melanin accumulation in appressoria, and is required for intracellular ROS homeostasis [108]. Of interest, the expression levels of *PEF1* (*Mdv1*) gene increased during appressorium formation, and its deletion resulted in reduced peroxisomes in appressoria [108]. Moreover, the elimination of one of the three isoforms of Pex11 in this fungus also affects virulence and appressorium development [119], suggesting that peroxisome fission is important for the development of this infection structure. It is worth noting that in *Colletotrichum orbiculare*, the causal agent of cucumber anthracnose, appressorium development is accompanied by ATG26-mediated pexophagy [120], suggesting that defective pexophagy might also contribute to the reduced invasiveness of the fission mutants (Section 7.2). As for *M. oryzae*, Dnm1 is required for full virulence in the corn smut fungus *Ustilago maydis* [121]. Additionally, the deletion of the fusion machinery gene *FZO1* also results in decreased infective growth and reduced appressoria penetration capability in *M. oryzae* [122].

Interestingly, in *A. fumigatus* the fission machinery is dispensable for virulence, whereas fusion dynamins are necessary [104]. Dimorphic human pathogenic fungi like *Cryptococcus neoformans* and *Candida albicans* also require the mitochondrial fusion machinery for full virulence. In *C. albicans*, the formation of hyphae is essential for infection, and an *mgm1* mutant strain is unable to form filamentous hyphae and presents sharply attenuated virulence [123]. In contrast, filamentous growth is only modestly reduced by defects on the fission machinery components in this yeast [124]. In *C. neoformans*, the deletion of the fusion gene *FZO1*, but not of any of the genes encoding proteins of the fission machinery, results in complete avirulence, which is associated with an increased susceptibility to ROS and a decreased survival inside macrophages [125]. The contrasting requirements for components of the fusion and fission machinery to achieve virulence in different pathogenic fungi suggest different contributions for mitochondrial dynamics to their distinct infection systems and remark the importance of mitochondria morphology in the development of fungal infection structures and virulence.

## 5. Sexual Development in Fungi Involves a Dynamic Regulation of Peroxisomes and Mitochondria

**Figure 3:**
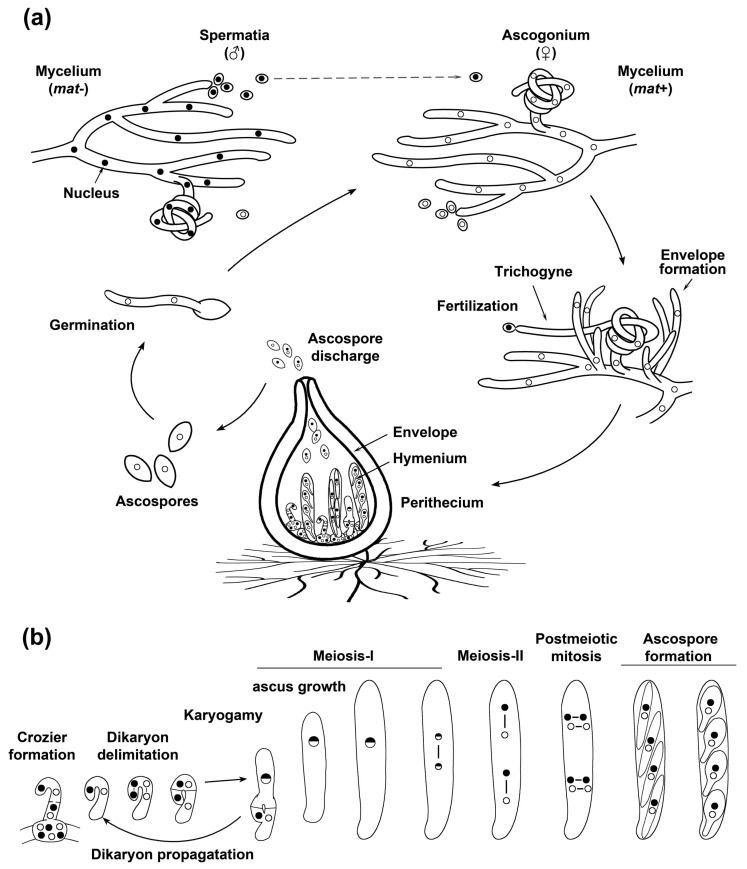
Sexual development of a model mycelial ascomycete. (**a**) The sexual life cycle of a heterothallic Sordariomycete. Mating in ascomycetes is controlled by a mating-type (*MAT)* locus, for which two alternative versions (idiomorphs) exist in heterothallic (self-sterile) species (denoted here as *mat+* and *mat-*). Strains of either mating type differentiate both, female gametangia (ascogonia) and male gametes (spermatia), which cross-fertilize when bearing opposite mating-type (opposite mating-type nuclei are illustrated as dots with different shading). Fertilization is attained by a specialized hypha—the trichogyne—which is produced by the ascogonium and that exhibits tropic growth towards a pheromone-producing male gamete. The ascogonium recruits neighboring hyphae, which develop a protective envelope around the ascogonium, producing a protoperithecium. Upon fertilization, the protoperithecium develops into a perithecium, and the fertilized ascogonium develops the hymenium, the fertile tissue where karyogamy, meiosis, and meiotic spore (ascospore) formation are accomplished. Ultimately, haploid mature ascospores are expelled out of perithecia and produce a new mycelium upon germination. (**b**) Hymenium development. Inside perithecia, the nuclei of the opposite mating type present in the fertilized ascogonia migrate into ascogenous hyphae that differentiate from the ascogonial cells. These ascogenous hyphae then develop into specialized hook-shaped cells, referred to as croziers, in which dikaryotic compartmentalization takes place. This process results from the synchronized mitoses (lines linking the dots represent spindles) of the two crozier leading nuclei, which are positioned in the crook part of the crozier and that differ in their mating type, and results in the formation of three cells separated by septa: an upper binucleated cell flanked by two uninucleate cells. The upper dikaryotic cell suffers karyogamy and enters meiosis at the same time that differentiates into an ascus (the meiocyte), whereas the two flanking uninucleate cells fuse to produce a new dikaryotic crozier, perpetuating the dikaryotic stage. In the example provided, which illustrates the development of *P. anserina*, asci then elongate from about 5 to more than 150 μm during the first meiotic prophase. After meiosis, a mitotic division results in the formation of eight nuclei, which are enclosed by pairs into four ascospores. Finally, ascospores grow and maturate inside the original ascus (for review, [126]).

Sexual reproduction in fungi is a complex developmental process that relies on the accurate progression of the sexual cycle stages—e.g., mating, karyogamy, and meiosis—that allow for the alternation of the haploid and diploid phases of the life cycle, and that enable genetic recombination (Figure 3 illustrates the sexual development of a model filamentous ascomycete). These processes are accompanied by the formation of multiple cell types and their differentiation involves important changes in cellular architecture and functioning. Moreover, in many fungi, sexual development takes place in complex multicellular structures and requires precise coordination between the development of the fertile tissue where sexual development takes place (the hymenium) and of the vegetative tissues that drive fruiting body morphogenesis [126]. This complex developmental process involves and relies on the dynamic regulation of peroxisome and mitochondrial activity.

### 5.1. Peroxisome and Mitochondrial Dynamics Are Regulated during Sexual Development

**Figure 4:**
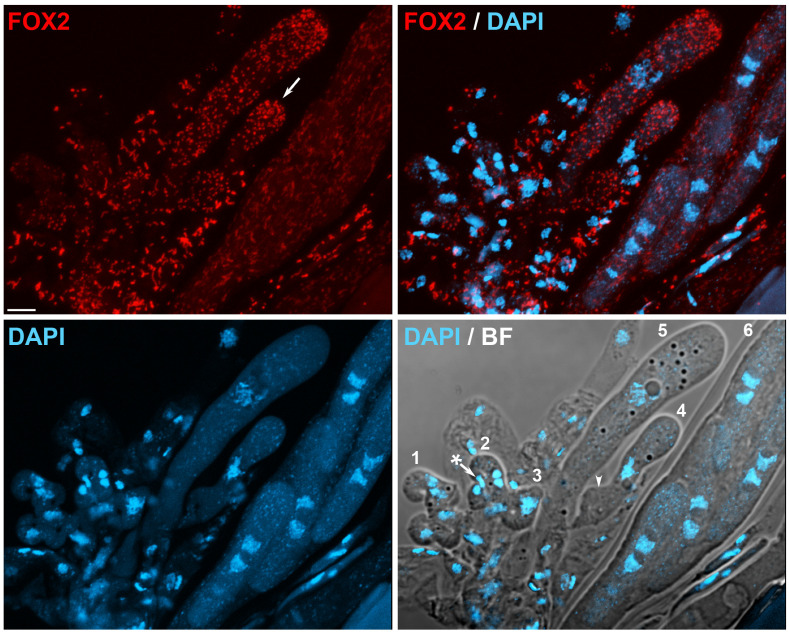
Peroxisome arrangement in representative stages of *P. anserina* sexual development. The numbering indicates successive developmental stages: (1) Young binucleate croziers. (2) Dikaryotic cell formation (note the lateral cell nucleus—asterisk—migrating into the basal cell to produce a new dikaryotic cell). (3) Karyogamy. (4–5) Asci at successive stages of meiotic prophase-I (arrow indicates peroxisomes concentrated at the ascus apex, arrowhead points to the reminiscent initial crozier cell). (6) Early ascospores. Peroxisomes were labeled with FOX2-mCherry. Nuclei and mitochondrial DNA (mtDNA) were stained by DAPI. BF, bright field. Scale bar, 5 μm. A section of this micrograph was previously reported in [102].

Meiosis and ascospore formation in the yeast *S. cerevisiae* involve dynamic changes in the arrangement and distribution of mitochondria, which are involved in promoting even segregation of mitochondria during the formation of ascospores and assuring mitochondrial inheritance during sexual reproduction. This process is detailed in Section 5.4. On the other hand, the analysis of peroxisome arrangement during the sexual development of *P. anserina* has shown that this process involves complex and defined regulation of peroxisome dynamics. On one side, the peroxisomes present in the hymenium sexual cells in this fungus are structurally different from those observed in the vegetative cells present within perithecia (e.g., paraphyses), which tend to be larger in the latter [127]. On the other side, peroxisomes also change morphologically during hymenium development, in correlation with the different developmental stages of the sexual cycle (Figure 4). Peroxisomes are relatively small and predominantly round-shaped in dikaryotic croziers (cell 1 in Figure 4). Mostly spherical peroxisomes also populate early asci (cells *4* and *5* in Figure 4); however, after asci reached their final length, peroxisomes change in shape and exhibit a more elongated morphology from the end of the first meiotic division until the early stages of ascospore formation (ascus *6* in Figure 4). Peroxisomes change again in shape along with ascospore differentiation, resulting in ascospores possessing numerous spherical peroxisomes [127]. Notably, progression through these developmental stages is also accompanied by changes in peroxisome number and distribution. Relatively scarce in dikaryotic cells, peroxisomes dramatically proliferate in the fast-growing asci along meiotic prophase-I (Figure 4, compare cells *1* to *5*), where, in addition, they are frequently concentrated towards the cell apex (Figure 4, arrow). The second proliferation of peroxisomes is observed during ascospore formation, which takes place following ascospore delineation and that produces grown ascospores crowded with peroxisomes before their maturation. Interestingly, the large number of peroxisomes present in these cells dramatically decreases upon maturation, probably through their elimination via pexophagy (see Section 7.3) [127,128]. These observations disclosed that meiotic development in *P. anserina* implicates a precise regulation of peroxisome dynamics.

### 5.2. Sexual Development Involves a Simultaneous Regulation of Peroxisome Dynamics and Biogenesis

Notably, in addition to peroxisome dynamics, the functional state of the translocation channel through which proteins are imported into peroxisomes is also regulated during meiotic development. Peroxisome matrix protein import is driven by two conserved import receptors—Pex5 and Pex7—which recognize the proteins to be imported into peroxisomes in the cytosol (by means of their peroxisome targeting signals PTS1 and PTS2, respectively) and target them to the organelle. The import receptors converge at the docking complex, which is formed by Pex13 and Pex14 (along with Pex17 and Pex14/17 in yeasts and filamentous fungi, respectively) and that interacts with a second peroxisome membrane complex—the RING-finger complex—to compose the docking/translocation machinery (or importomer) [129]. Following docking, the import receptors are inserted into the peroxisome membrane and after releasing their cargoes they are cycled back to the cytosol. Although the precise mechanism of protein translocation is unknown, the receptor Pex5 plays a central role in this process. This protein could act as a translocator that inserts into the docking/translocation machinery to release its cargo [129,130]. Alternatively, Pex5 could constitute by itself a core component of a transient translocation channel. It has been postulated that PTS1 and PTS2 proteins in *S. cerevisiae* are imported into peroxisomes through two independent channels, which are, respectively, conformed by Pex5 and by the PTS2 co-receptor Pex18, a Pex7 accessory protein [131,132,133]. The export of the import receptors is driven by the receptor export machinery (or exportomer). This complex consists of ubiquitination and dislocation subcomplexes, which prime the receptor for recycling by ubiquitination and dislocate it from the peroxisome membrane, respectively [133,134]. A central element of the docking/translocation machinery is the peroxin Pex14, which constitutes a core component of the peroxisome protein translocation channel. Consistent with a central role for Pex14 in *P. anserina*, this protein is required for both PTS1 and PTS2 import pathways in hyphae. However, this protein is not absolutely required during meiotic development and is dispensable for import at the ascus growth phase during meiotic prophase I, as well as at the early stages of ascospore formation. Furthermore, the docking peroxin Pex14/17 is also not required for import at the same developmental stages [135]. These observations indicate that an import-competent translocation channel can be assembled in absence of Pex14 at specific developmental stages and suggest that the constitution of the translocation channel is modulated during meiotic development.

In addition, different stages of meiotic development in *P. anserina* rely on different configurations of the peroxisome protein import system. In this fungus, removal of any peroxin of the RING-finger complex or the exportomer, as well as the elimination of the docking peroxin Pex13 or of Pex8—the peroxin that connects the docking and the RING-finger complexes—blocks meiotic development initiation [128,135,136,137]. The sexual development of mutant strains deficient for any of these peroxins is blocked at the dikaryotic stage, and their dikaryotic crozier cells do not undergo karyogamy or enter meiosis. Instead, dikaryotic cells divide mitotically and engage in the formation of new croziers. Consequently, asci and ascospores are never produced. Conceivably, these defects are associated with a failure to induce meiosis (reviewed in [77]). Unexpectedly, this phenotype is not observed when either or both import receptors—Pex5 and Pex7—are missing, nor upon elimination of Pex14 or Pex14/17 [135,138]. However, the PTS2 co-receptor Pex20 is also absolutely required for meiotic induction [135]. Moreover, the single replacement of a Pex20 cysteine, which is the potential target residue for the monoubiquitination that drives Pex20 recycling, also impedes meiotic initiation, suggesting that, as an import receptor, Pex20 recycling from peroxisomes is essential for meiotic induction [137]. These findings suggest that the initiation of meiotic development in *P. anserina* relies on a Pex20-driven import pathway. Consistent with this notion, Pex20 has recently been found to act as an import receptor for a non-PTS1/non-PTS2 protein in the yeast *Yarrowia lipolytica* [139]. Importantly, while the import receptors Pex5 and Pex7 are dispensable for meiotic induction, their elimination results in altered ascospore nuclear distribution, suggesting defective nuclear progression during meiotic development [138]. These findings suggest that meiotic initiation and progression in *P. anserina* depend on distinct configurations of the peroxisome protein translocation channel, which selectively conduct the import of proteins that contribute to different stages of meiotic development. Notably, the developmental stages that display different import requirements in *P. anserina* are correlated with the phases of meiotic development that show changes in peroxisome dynamics, suggesting a concerted regulation of the proteins that govern peroxisome dynamics and assembly during meiotic development.

### 5.3. A Possible Interplay between Peroxisomes and Mitochondria during Sexual Development

The above observations indicate that peroxisomes perform different functions during *P. anserina* meiotic development, which are associated with a developmental regulation of the biogenesis and dynamics of this organelle. Moreover, evidence also indicates that this regulation differentially impacts the activity and dynamics of mitochondria. In this fungus, loss of the import receptor Pex5 or Pex7 results in mitochondrial abnormalities in vegetative hyphae that are consistent with altered mitochondrial dynamics. Specifically, loss of Pex5 resulted in mitochondria that were predominantly round-shaped and sometimes aggregated, in contrast to the elongated morphology of wild-type mitochondria. In addition, the elimination of Pex7 produced mitochondria that were thinner and larger than normal [138]. Of interest, these defects were not observed in absence of Pex2, a peroxin of the RING-finger complex that is required for meiotic induction. These observations indicate that the failure of peroxisome function generated in absence of Pex5 or Pex7 echoes on mitochondrial dynamics and suggests that mitochondria could contribute to the developmental abnormalities produced by the loss of these import receptors. Moreover, since the loss of Pex5 and Pex2 produced different developmental outcomes, these finding also suggests that different peroxisome configurations, with distinct developmental impacts, establish differential interactions with mitochondria.

### 5.4. Peroxisome and Mitochondrial Dynamics Are Required for Organelle Segregation during Sexual Development

Essential for sexual reproduction is the inheritance of mitochondria, organelles that cannot be produced de novo and that rely on an accurate segregation process that ensures their partitioning into the meiotic offspring. Research in the yeast *S. cerevisiae* has given insights into the mechanism governing mitochondrial inheritance during meiosis. From the pre-meiotic S-phase, mitochondria are anchored at the cell cortex by the MECA complex (see Section 3.1) [140,141,142] until anaphase II detachment, promoted by the continuous disassembly of MECA by Ime2 phosphorylation [142]. Afterward, detached mitochondria are redistributed to the perinuclear area, and they remain associated with the dividing nucleus until ascospore delineation [140,141]. The regulated detachment of mitochondria and their subsequent association to the nuclear membrane may be essential for mitochondrial segregation into developing spores [142]. Additionally, mitochondrial severing by the fission machinery upon ascospore formation is critical for the even partitioning of both mitochondria and mitochondrial DNA (mtDNA) during this process. Interestingly, following ascospore delineation, mitochondria present in these cells further divide upon tetrad maturation, in a process that does not depend on the fission proteins Dnm1, Mdv1, or Fis1. This indicates that an alternative mitochondrial fission process operates during ascospore differentiation in *S. cerevisiae* [140].

**Figure 5:**
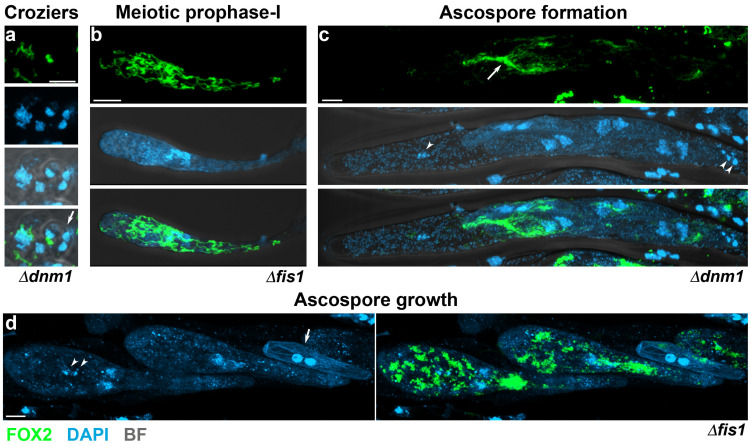
Contribution of the fission machinery to peroxisome dynamics during *P. anserina* sexual development. Examples of the effect of abrogating the fission machinery during sexual development: (**a**) Croziers. Visible are a dikaryotic tetra-nucleate crozier (right, arrow points to the dikaryotic cell, which lacks peroxisomes) and a crozier following karyogamy (left). (**b**) First meiotic prophase ascus. (**c**) Early ascospores. Four binucleate ascospores inside an ascus are visible; note the asymmetric distribution of peroxisomes and the cluster of elongated peroxisomes (arrow) that failed to be incorporated into ascospores. (**d**) Growing ascospores. Visible are two normal ascospores that have differentiated a large head and a slender tail, and a small aberrant ascospore (arrow, note the very reduced number of peroxisomes of this ascospore). Arrowheads in (**c**,**d**) point to mtDNA clusters. Cells were issued from homozygous sexual crosses of *∆dnm1* or *∆fis1* mutants, which exhibit a very similar phenotype. Please refer to Figure 4 to appreciate the morphology of wild-type peroxisomes at the equivalent stages. Peroxisomes were labeled with FOX2-GFP. Nuclei and mtDNA were stained by DAPI. BF, bright field. Scale bar, 5 μm.

In contrast to *S. cerevisiae*, the elimination of the fission proteins Fis1 or Dnm1 did not markedly affect mitochondria segregation during *P. anserina* sexual development. As in other organisms, Dnm1 and Fis1 were required for proper mtDNA distribution in this fungus, and their elimination resulted in the clustering of mitochondrial nucleoids during sexual development (illustrated in Figure 5, arrowheads). Nonetheless, mtDNA segregation was also not noticeably affected during this process [102]. Interestingly, however, Fis1 and Dnm1 were required for peroxisome segregation at two key developmental stages of sexual development. During the dikaryotic stage of *DNM1-* or *FIS1-*deleted mutants, peroxisomes present in crozier cells were often elongated and asymmetrically distributed. Actually, several croziers of these mutants, or the dikaryotic cells present in them, contained few, or even lacked, detectable peroxisomes (Figure 5a). In correlation with this finding, several early asci contained small punctate peroxisomes in small numbers, instead of the networks of elongated peroxisomes present in most asci in these mutants (Figure 5b). These asci were interpreted as asci derived from crozier dikaryotic cells that failed to inherit peroxisomes, and their punctate peroxisomes as peroxisomes produced de novo in the ascus [102]. Large networks of elongated peroxisomes with asymmetrical distribution were observed in asci lacking Fis1 or Dnm1 from late meiosis until the stages anteceding ascospore formation (Figure 3). This abnormal arrangement resulted in peroxisomes that failed to be incorporated into ascospores, and in uneven peroxisome partitioning during ascospore formation (Figure 5c) [102]. These observations indicate that the segregation of peroxisomes during the differentiation of asci and ascospores in *P. anserina* relies on the function of the peroxisome-mitochondrial fission machinery. The finding that the fission machinery was not required for mitochondria segregation indicates different constraints for the segregation of these two organelles, even when their dynamics are regulated by common key factors. These differences might reflect alternative processes involved in more stringently controlling the segregation of mitochondria, organelles that cannot be produced de novo.

### 5.5. Peroxisome and Mitochondrial Dynamics Are Required for Sexual Development

The inhibition of the fission of mitochondria and peroxisomes produces multiple defects during sexual development in mycelial ascomycetes. In *A. nidulans*, the elimination of DnmA (Dnm1) or FisA (Fis1) results in the formation of large numbers of Hülle cells [100]—specialized nursing cells for sexual fruiting body (cleistothecium) development, which serve as storage of the parental genetic information during *Aspergilli* development [143]—which was considered as indicative of premature sexual development initiation [100]. In contrast, elimination of Dnm1 or Fis1 delayed sexual development in *P. anserina*. In addition, two key stages of sexual development in this fungus—karyogamy and ascospore differentiation—require the function of these proteins. In *P. anserina*, the deletion of *DNM1* or *FIS1* resulted in several early differentiating asci, which contained unfused nuclei at stages where karyogamy has normally taken place [102]. This finding is consistent with the defective peroxisome inheritance of *fis1* and *dnm1* mutant dikaryotic crozier cells, and with the requirement of peroxisomes in these cells for karyogamy and meiotic induction [135] (see Section 5.2). Still, this karyogamy delay was moderate, which probably reflects that peroxisomes could be produced de novo in the ascus [102]. More drastically affected by *DNM1* or *FIS1* deletion was the differentiation of ascospores. In *P. anserina*, following their delimitation, ascospores grow asymmetrically to increase their volume about 10 times and differentiate a globular head and a narrow tail (Figure 3b). Whereas the tail is separated by a septum and degenerates to produce a primary appendage, the head develops into the mature ascospore [144]. A large number of asci containing small ascospores, as well as very small aberrant ascospores, which did not differentiate head and tail cells (Figure 5d, arrow), were produced in this fungus when Fis1 or Dnm1 were missing. Genetic and cytological analyses indicated that these proteins are required for a very early stage of ascospore formation, occurring after meiotic nuclei segregation and before ascospores become autonomous. Furthermore, the aberrant undifferentiated ascospores were in most cases associated with highly aberrant clusters of tightly packed mitochondria, which were not observed in normal mutant ascospores, and they very frequently contained very limited numbers of peroxisomes (Figure 5d) [102]. These observations show that defective ascospore differentiation is correlated with severe defects in the dynamics of both peroxisomes and mitochondria, suggesting a complex role and interplay for peroxisome and mitochondrial dynamics in this process. Importantly, in keeping with a conserved function for the mitochondrial-peroxisome fission proteins in sexual development, the cleistothecia produced by *dnmA* and *fisA* mutants in *A. nidulans* accumulated sterile ascogenous tissue and produced reduced numbers of viable ascospores [100]. Moreover, the deletion of *dnm1* in *N. crassa* produces sterility [109]. Notably, in this fungus, the ER-Mitochondria Encounter Structure (ERMES) is also required for sexual development. Mitochondrial fission relies on the establishment of mitochondrion-ER contact sites, which in fungi are mediated by the ERMES complex [145]. In *N. crassa*, mutations in the *mmm-1* gene, which codes for the ERMES protein Mmm-1, also result in sterility, which is characterized by altered perithecium morphogenesis and incapacity to produce ascospores [146]. The ERMES complex is associated with mitochondrial fission sites in *S. cerevisiae* [147]. Thus, the above observations could indicate that the ER promotes mitochondrial fission necessary for sexual development. Nonetheless, since the ERMES complex is implicated in additional functions, including lipid transfer, mitochondrial morphology, and mitochondrial and mtDNA inheritance [148], further research is required to better understand the role of the ERMES complex in *N. crassa* sexual reproduction.

### 5.6. Mitochondrion-Associated Regulatory Systems Control Sexual Development in Fungi

In line with the critical roles performed by mitochondria during sexual development, and with the involvement of developmental regulation of mitochondria dynamics during this process, cellular mechanisms that coordinate mitochondrial activity with the regulatory systems that control sexual reproduction could exist. Evidence for the existence of such regulatory systems has been provided in ascomycete fungi.

#### 5.6.1. Mitochondria Is Involved in the Regulatory Network Controlling Sexual Development

The high-mobility-group (HMG)-box protein mtHMG1 is a mitochondrial protein that is essential for sexual reproduction in *P. anserina* [149,150]. This protein is required both for the development of the vegetative tissues during perithecium formation, as well as for the development of the hymenium. The participation of this protein in sexual reproduction in Pezizomycotina fungi seems to be conserved, as the *A. nidulans* orthologous protein HmgB is also involved in sexual development [151,152]. Although the precise function of these proteins in this process is not fully understood, part of their function is likely related to mtDNA maintenance. However, they seem to perform additional functions. While it predominantly localizes to mitochondria, a fraction of HmgB is located in nuclei in *A. nidulans*, and putative nuclear localization signals are present in *P. anserina* mtHMG1 [149,152]. Furthermore, mtHMG1 takes part in a regulatory network of HMG-box transcription factors, which governs the expression of mating-type genes and regulates sexual development in *P. anserina* [149]. These findings suggest that mtHMG1 is involved in a transcriptional regulatory circuit that controls sexual development in ascomycetes, and might be crucial for connecting this system to mitochondria function and maintenance.

#### 5.6.2. A Mitochondrial Check Point Controls the Progression of Sexual Development

In *P. anserina*, mutations of the *CIT1* gene, which encodes for mitochondrial citrate synthase, inhibit meiotic progression beyond the first meiotic division. In this fungus, the deletion of *CIT1* produces a discrete arrest at a defined stage of meiosis—the diffuse stage—whereas it does not noticeably affect vegetative growth. Moreover, genetic and biochemical analyses of distinct randomly obtained *cit1* mutant strains suggested that the presence of citrate synthase itself, rather than its enzymatic function, appears to be determinant for meiotic progression [153]. This research supported the existence of a regulatory system that inhibits meiotic progression when citrate synthase is missing. Of note, in addition to the tricarboxylic acid cycle, citrate synthase is involved in the glyoxylate cycle, and this enzyme potentially localizes to both mitochondria and peroxisomes in *A. nidulans*, where its elimination also precludes ascospore formation [154]. It is thus tempting to speculate that this regulatory system could have a role in sensing the status/metabolic interplay between peroxisomes and mitochondria. Actually, the original *P. anserina CIT1* mutations that disclosed this regulatory system were initially identified as suppressor mutations of a metabolic defect of peroxisome biogenesis-defective *pex2* mutants [153,155].

## 6. Woronin Bodies, Specialized Peroxisome-Derived Organelles Important for Fungal Development

A remarkable example of the specialization of an organelle associated with the lifestyle of a eukaryotic organism is provided by the Woronin bodies. Hyphae from filamentous basidiomycetes and ascomycetes are divided by septa with a central pore, which permits the generation of hyphal compartments and simultaneously allows the exchange of cytoplasmic content, such as metabolites, organelles, or even genetic material, between adjacent compartments. The perforated septum in these phyla is sealed by different structures, which dynamically control the protoplasmic flow across the pore and prevent the massive loss of cytoplasmic content during mechanical damage. In addition, it plays a relevant role in the processes of cellular differentiation. The basidiomycete pore is delimited by bell-shaped septal swellings, which are surrounded by specialized ER-derived membranes known as the septal pore cap (SPC), defining an arrangement known as the dolipore septum [156,157]. In contrast, the cytoplasmic stream through the pore in ascomycetes is controlled by a specialized organelle stemming from peroxisomes known as the Woronin body, which functions as a plug [156,158,159,160].

Woronin bodies are characterized by the Hexagonal peroxisome (Hex) protein, which self-assembles into rigid oligomers that constitute the core of these organelles. Hex1 has a PTS1 sequence and is initially imported into peroxisomes, where is sorted to the organelle cortex by its membrane receptor WSC (Woronin Sorting Complex), producing asymmetrically distributed Hex1 aggregates. WSC then interacts with the cytoplasmic protein Leashin, which is associated with the cell cortex, to promote Woronin body segregation and budding from peroxisomes, in a process that requires the peroxin Pex11 (reviewed in [156]).

Early analyses of the subcellular organization during fungal development showed that Woronin bodies are associated with the differentiation of distinct cell types in conidiation (e.g., [79]) (Figure 2c,d), as well as to specific sexual development-associated cell types [161], suggesting that Woronin body function is important for fungal development. An involvement for Woronin bodies during conidiation has been exposed in *Fusarium graminearum (Gibberella zeae)*, a devastating pathogen of cereals, where either the deletion or overexpression of *HEX1* resulted in a significant reduction in conidiation [162]. Similarly, loss of Woronin bodies results in reduced conidiation in the nematophagous *A. oligospora* [163], in *Aspergillus flavus* [164] and the entomopathogenic *M. robertsii* [165], whereas in *A. nidulans* [166] and *N. crassa* [160] it has a minor impact in conidiation. Moreover, no visible conidiation defects are produced by Hex1 elimination in fungi like *M. oryzae* [167] and *A. fumigatus* [168]. Of note, the absence of HexA in *Aspergillus oryzae* produces a complete lack of conidiation in hypotonic conditions [169], suggesting that Woronin bodies are critical to maintaining hyphal integrity and that a perturbation in this process could have important detrimental developmental repercussions.

In the nematophagous fungus *A. oligospora*, *HEX1* transcription is regulated during trap cell formation in a StuA-dependent manner, and Woronin bodies are lost when this master transcriptional regulator of trap cell differentiation is missing, showing that the regulation of Woronin body formation is part of the transcriptional program that regulates trap cell formation in this fungus [99]. Moreover, elimination of Woronin bodies in this fungus by deleting the *Hex1* gene results in complete incapacity to produce trap cells and to capture nematodes, showing that Woronin bodies are essential for the predacious development of this fungus [163].

On the other hand, Woronin body function is also required for proper appressorium formation and virulence both in plant and in insect pathogenic fungi. In *M. oryzae*, appressoria produced by *hex1* mutants display morphological abnormalities and highly reduced capacity to produce penetration pegs and infect plants [167]. The infection capacity of *M. robertsii* is also reduced upon elimination of Hex1, in correlation with impaired production of appressoria [165]. Similarly, loss of Hex1 also reduced virulence of *F. graminearum*, a fungus that does not rely on appressoria to infect its host plants [162].

A possible link between Woronin bodies and sexual development was disclosed by the characterization of the protein PRO40 in the model ascomycete *Sordaria macrospora*. PRO40 is a WW domain-containing protein that serves as a scaffold for the cell wall integrity mitogen-activated protein kinase signaling pathway, and that is essential for perithecia formation [170]. In this fungus, PRO40 localizes to septal plugs and partially colocalizes with HEX1. Furthermore, at least a fraction of this protein resides in Woronin bodies [171]. However, the biogenesis and integrity of Woronin bodies are not affected by the absence of PRO40, and additional cellular processes, which are controlled by the cell wall integrity pathway—including hyphal fusion and growth—depend on this protein [170,171]. Thus, the relevance of the association of this signaling factor to Woronin bodies during sexual development remains unclear.

Altogether, the above observations indicate that the dynamic regulation of peroxisomes during developmental processes in fungi includes the differentiation of the specialized Woronin bodies, which likely participate in regulating the integrity, connectivity, and dynamic compartmentalization of differentiated cells during development.

## 7. Fungal Development Also Relies on the Autophagic Removal of Mitochondria and Peroxisomes

Cell differentiation implicates important changes in cell functioning, which frequently involve a substantial metabolic reorganization, and drastic organelle remodeling and renewal. Autophagy is a catabolic process that allows for the clearance of intracellular components. This process allows for the recycling of the degradation products, and permits the elimination of dysfunctional subcellular structures, constituting a fundamental system for intracellular quality control [34]. This process participates in regulating the activity of peroxisomes and mitochondria by selectively removing them at defined developmental events and is determinant for the efficient progression of diverse developmental processes in fungi.

### 7.1. Mitophagy and Pexophagy Are Required for Asexual Sporulation

Tracing autophagy during the conidiation of *M. oryzae* by visualizing Atg8 revealed that autophagy is significantly induced during the differentiation of the distinct cell types involved in conidiation, including aerial hyphae, conidiophores, and conidia [172]. Similarly, autophagy was detected during conidiophore and conidia formation in *A. oryzae* [173], as well as in both *B. bassiana* asexual spores, conidia and blastospores [174], showing that autophagy naturally occurs during the formation of asexual spores of diverse fungi. Consistent with an important role for autophagy in these processes, the abrogation of core autophagic machinery components compromise asexual spore formation in numerous fungi [175,176]. Notably, mitophagy has also been detected during *M. oryzae* conidiation, where it specifically takes place in the foot cells that originate aerial hyphae and not in the latter, showing a high degree of specialization and compartmentalization of mitophagy during fungal development [177]. This process was found to depend on the sorting nexin Atg24, which is required for mitophagy in this fungus but not for pexophagy or nonselective autophagy. Moreover, the genetic elimination of this protein resulted in a significant decrease in conidiation, which was characterized by a reduction in aerial hyphae growth, a decreased conidiophore formation, and a strong reduction in conidia formation. Interestingly, while no clear orthologue of the yeast mitophagy receptor Atg32 is present in *M. oryzae* (nor other filamentous fungi), the introduction of a *S. cerevisiae ATG32* allele into the *M. oryzae atg24* mutant partially restored conidia formation. These observations suggest that Atg24 acts as a mitophagy receptor in *M. oryzae*, and are consistent with the notion that the conidiation defects generated by the loss of Atg24 are associated with a mitophagy deficiency [177]. Akin to *M. oryzae,* deletion of *ATG24* results in decreased aerial mycelium formation in *P. anserina* [178] and in *F. graminearum*, as well as in a strong reduction in conidia formation in *F. graminearum* [179]. Similarly, the number of microconidia in *P. anserina* was dramatically reduced upon *ATG24* deletion (see Section 7.3) [178]. Nonetheless, in addition to mitophagy, Atg24 also participates in pexophagy and non-selective autophagy in *P. anserina*, and its contribution to selective autophagy in *F. graminearum* remains to be established. Therefore, the actual contribution of mitophagy and pexophagy to these developmental processes requires further analysis.

In contrast to mitophagy, pexophagy was not detectable during *M. oryzae* conidiation, and the analysis of factors implicated in pexophagy in this fungus indicated that this process is dispensable for conidiation [180]. However, evidence that pexophagy participates in fungal conidiation has been provided by *Colletotrichum orbiculare*—the causal agent of cucumber anthracnose. In this ascomycete, the elimination of Atg26, a sterol glucosyltransferase required for pexophagy, generates a significant reduction in conidiation [120]. Conidiation is also reduced in *A. oryzae* upon elimination of Atg26, nevertheless, evidence indicates that this protein is involved in the autophagic degradation of peroxisomes, mitochondria, and nuclei in this fungus [181].

Consistent with the participation of mitophagy and/or pexophagy during the conidiation of *B. bassiana,* the elimination of the selective autophagy adapter Atg11 in this fungus also significantly reduces conidiation [182]. Atg11 is required for pexophagy and mitophagy in this fungus. Moreover, vacuolar degradation of peroxisomes, but not mitochondria, was observed in aerial hyphae during asexual development, and this process was inhibited upon loss of Atg11, suggesting a more prominent role for pexophagy than mitophagy during conidiation. Nonetheless, Atg11 is known to participate in additional selective autophagy pathways in fungi, including ER-phagy and the cytoplasm-to-vacuole targeting (Cvt) pathway [37], therefore, further examination is required to ascertain the actual contribution of these processes during *B. bassiana* conidiation. The autophagy selectivity provided by Atg11 in distinct fungi differs. In *A. oryzae* Atg11 is required for pexophagy and mitophagy, but dispensable for the Cvt pathway [183], whereas in *Acremonium chrysogenum* it is also required for the Cvt pathway and likely participates in bulk autophagy [184]. Furthermore, the developmental outcome of Atg11 elimination also differs among fungi. In contrast to *B. bassiana, ATG11* deletion did not affect the conidiation of *A. oryzae* [183], while enhanced that of *A. chrysogenum* [184] and partially reduced it in *F. graminearum* [179]. Moreover, while required for conidiation in *B. bassiana*, Atg11 is dispensable for blastospore formation in this fungus [182]. These observations disclosed notable differences in the regulation of selective autophagy among different fungi and suggest distinct requirements for mitophagy and pexophagy during the different asexual differentiation processes of fungi.

### 7.2. Mitophagy and Pexophagy Are Required for Pathogenic Development in Fungi

Autophagy has also been associated with and plays a critical role in, the pathogenic development of fungi [176,185]. In *M. oryzae*, appressorium formation and maturation depend on the autophagic death of the conidium from where the appressorium develops, and on the delivery of the conidium contents into the developing appressorium [186]. A genome-wide analysis of autophagy-related genes in this fungus revealed that loss of any protein of the core autophagy machinery inhibits conidial cell death during appressorium development and impairs appressorium maturation and function. In contrast, none of the proteins predictably involved in selective autophagy was required for appressorium-mediated plant infection [187], arguing against a role for pexophagy or mitophagy in this process. Nonetheless, further examination of mitophagy in *M. oryzae* revealed that the loss of Atg24 results in delayed appressorium infectivity and reduced growth of the invasive hyphae. In line with these findings, Atg24-dependent vacuolar degradation of mitochondria was observed during the initial stages of the plant infection [122]. These observations disclosed that Atg24-driven mitophagy is required for the establishment of the blast disease.

On the other hand, analysis of pexophagy in *M. oryzae* demonstrated that this process takes place at different stages of appressorium development, including the germinating conidium, the developing appressorium, and the infective hyphae produced by appressoria. However, consistent with previous findings, the genetic analysis of the factors required for pexophagy showed that this process is not essential for appressorium development [180]. In contrast, the appressorium-mediated infection of *C. orbiculare* relies on pexophagy [120]. In this fungus, the cellular integrity of appressoria produced by an *ATG26* deletion mutant strain is disturbed, and they fail to produce infectious hyphae. In *C. orbiculare*, peroxisomes are successively degraded at the progressive stages of appressorium development. Peroxisomes are removed by autophagy from appressoria upon maturation, prior to the invasion process, in a process that depends on Atg26. These findings demonstrate that pexophagy has an important role during the development of this infection structure [120]. In *F. graminearum*, virulence is reduced, to a different extent, upon elimination of several genes predictably involved in mitophagy and/or pexophagy, including *ATG11, ATG24, ATG26, ATG33,* and *ATG37* [179]. Nonetheless, the precise function of the corresponding proteins in *F. graminearum* awaits elucidation.

### 7.3. Sexual Development Relies on Pexophagy

Autophagy also plays major roles during sexual development in fungi [179,188,189,190,191,192]. During sexual reproduction, a developmental specific removal of peroxisomes takes place during ascospore development in *P. anserina*. In this fungus, peroxisomes proliferate during ascospore differentiation, to then be significantly removed upon ascospore maturation [127]. Although the actual involvement of pexophagy in this process has not been demonstrated, the elimination of Atg24 in *P. anserina* causes a delay in the maturation of ascospores (issued from heterozygous crosses, see below) [178], suggesting a role for pexophagy in this process. Notably, loss of Atg24 also results in sterility in *P. anserina*, which is associated both, with a reduced capacity of the *atg24* mutants to serve as female partners and, more prominently, to an inability to produce spermatia (male gametes). Nonetheless, since *P. anserina* Atg24 is required for pexophagy, mitophagy, and non-selective autophagy [178], further research is needed to understand the actual contribution of these processes during *P. anserina* sexual development.

Like for the yeast mitophagy receptor Atg32, no orthologues of the pexophagy receptors Atg30 or Atg36 are found in filamentous fungi. In contrast, orthologues of the mammalian pexophagy receptor NBR1 are conserved throughout fungi. NBR1 is required for pexophagy in the ascomycete *S. macrospora*, and gene replacement experiments have shown that its activity in this process can be conducted, to a significant extent, by the human NBR1 protein, showing that NBR1 constitutes a pexophagy receptor in mycelial ascomycetes [193]. Interestingly, NBR1 is required for sexual development in *S. macrospora*. In this fungus, the formation of ascogonia, protoperithecia, and perithecia is delayed and significantly reduced upon *NBR1* deletion. Moreover, the asci produced by the *nbr1* mutant perithecia are defective and they rarely develop mature ascospores [193]. These findings indicate that pexophagy is required for the later stages of sexual development in this fungus. Indicating different pexophagy requirements during sexual development of distinct fungi, loss of the sorting nexin Atg20 in *F. graminearum*, which is involved in pexophagy, the Cvt pathway, and non-selective autophagy in this fungus, did not affect sexual development [194].

### 7.4. Roles of Mitophagy and Pexophagy during Fungal Development

The cellular functions underlying the developmental defects produced by defective pexophagy and mitophagy remain poorly understood. Most fungal structures affected by unpaired mitophagy or pexophagy are non-assimilating structures, and their development conceivably involves the utilization of nutrients derived from the recycling of organelles via autophagy. Consistent with this interpretation, *S. macrospora nbr1* pexophagy mutants are more susceptible to starvation than a wild-type strain [193], and the addition of exogenous nutrients to the cultures of *B. bassiana atg11* mutants partially alleviated their conidiation defects [182]. However, in contrast to this developmental process, the addition of glucose did not improve pathogenesis in *C. orbiculare atg26* mutants [120]. On the other hand, compromised pexophagy or mitophagy could lead to imbalanced ROS formation and deregulate redox homeostasis. The perithecium-formation defects of the *S. macrospora* pexophagy-deficient *nbr1* strains were exacerbated upon the addition of H_2_O_2_ [193]; the loss of either, the selective autophagy adapter Atg11 in *B. bassiana* [182], or the sorting nexin Atg24 involved in mitophagy in *M. oryzae* [177], resulted in increased sensitivity to menadione-induced oxidative stress, indicating an increased sensitivity to mitochondrial ROS in these latter mutants. Furthermore, the addition of antioxidants to *M. oryzae atg24* mutant cultures produced a mild restoration in their conidiation [177]. On the other hand, an important role for autophagy is the removal of excess or dysfunctional organelles, which allows the cell to renew its components and to control its quality and proper functioning. In *P. anserina*, the large proliferation of peroxisomes that accompanies ascospore differentiation is associated with ascospore melanization, where they likely provide melanin biosynthetic precursors issued from the β-oxidation of fatty acids [77,128,195]. This pathway produces large amounts of H_2_O_2_, which could potentially be harmful to peroxisomes; thus, the elimination of peroxisomes observed during ascospore maturation could be required to remove damaged peroxisomes, and to adjust peroxisome constitution for the subsequent developmental stage. This clearance process could have a role in ascospore rejuvenation prior to germination [127]. In line with this hypothesis, the lifespan of mycelia issued from Atg24-deficient ascospores in *P. anserina* is reduced [178]. Although further research is required to better understand the developmental roles of pexophagy and mitophagy, the above observations suggest multiple contributions for these processes in distinct fungal developmental processes. These findings also indicate that the developmental functions of mitophagy and pexophagy are not widely conserved among fungi and stress the relevance of performing comparative studies in fungi differing in their evolutionary lineage and lifestyle.

## 8. Concluding Remarks

The development of fungi involves the orderly progression of multiple cellular events, which include cell division processes, differentiation of multiple cell types, and morphogenesis of complex multicellular structures. Early research on mitochondrial and peroxisome function in fungi revealed that these organelles are critical for fungal development. Recent studies have shown that, in addition to their well-known metabolic functions, the regulation of the dynamics of these organelles also plays important roles in fungal development. Developmental processes in fungi involve a dynamic regulation of the proliferation, distribution, segregation, morphology, and removal of these organelles, and the perturbation of some of these processes has important repercussions in the establishment and progression of different developmental processes. Remarkably, for many of these processes, mitochondrial and peroxisome dynamics are governed by common factors, underscoring the intimate interplay maintained by these organelles in the cell, and suggesting a concerted activity of these organelles during fungal development. Nevertheless, despite a large body of information implicating peroxisome and mitochondrial dynamics in numerous developmental events, the actual functions underlying many of these developmental implications are largely unknown. Moreover, there is still limited information about the orchestrated regulation of peroxisome and mitochondrial dynamics during development, and for many developmental processes that rely on common peroxisome/mitochondrial regulatory factors, the contribution of each organelle is ambiguous. Further comparative research is required to better understand the regulation and the diverse developmental implications of mitochondrial and peroxisome dynamics in fungi. However, gathered information has underscored the complex and important roles associated with these processes among the diversity of the fungal developmental processes.
